# Unraveling the regulatory connections between two controllers of breast cancer cell fate

**DOI:** 10.1093/nar/gku360

**Published:** 2014-05-03

**Authors:** Jinho Lee, Abhinav Tiwari, Victor Shum, Gordon B. Mills, Michael A. Mancini, Oleg A. Igoshin, Gábor Balázsi

**Affiliations:** 1 Department of Systems Biology – Unit 950, The University of Texas MD Anderson Cancer Center, Houston, TX 77054, USA; 2 Department of Bioengineering, Rice University, Houston, TX 77005, USA; 3 Department of Physics, University of Houston, Houston, TX 77004, USA; 4 Department of Molecular and Cellular Biology, Baylor College of Medicine, Houston, TX 77030, USA

## Abstract

Estrogen receptor alpha (ERα) expression is critical for breast cancer classification, high ERα expression being associated with better prognosis. ERα levels strongly correlate with that of GATA binding protein 3 (GATA3), a major regulator of ERα expression. However, the mechanistic details of ERα–GATA3 regulation remain incompletely understood. Here we combine mathematical modeling with perturbation experiments to unravel the nature of regulatory connections in the ERα–GATA3 network. Through cell population-average, single-cell and single-nucleus measurements, we show that the cross-regulation between ERα and GATA3 amounts to overall negative feedback. Further, mathematical modeling reveals that GATA3 positively regulates its own expression and that ERα autoregulation is most likely absent. Lastly, we show that the two cross-regulatory connections in the ERα–GATA3 negative feedback network decrease the noise in ERα or GATA3 expression. This may ensure robust cell fate maintenance in the face of intracellular and environmental fluctuations, contributing to tissue homeostasis in normal conditions, but also to the maintenance of pathogenic states during cancer progression.

## INTRODUCTION

Transcription factors (TFs) form regulatory networks playing major roles in cell fate determination, from bacteria to mammalian cells (1). Transcriptional regulation in microbes is relatively well understood and several classical examples of cell fate-controlling networks have been unraveled, including the lambda switch (2) or lac operon (3) in *Escherichia coli*, sporulation decision in *Bacillus subtilis* (4) or the galactose uptake network in yeast (5). Unfortunately, the molecular details of transcription in mammalian cells are much less understood and some networks underlying crucial cell fate decisions such as Human Immunodeficiency Virus (HIV) latency (6) or stem cell differentiation (7,8) are still being uncovered. A particularly important area requiring more research is cell fate determination in cancer (9,10). The clonal evolution theory of cancer drove efforts to identify cancer-related mutations in ever increasing detail (11). However, not all cancer subtypes are associated with particular mutations and the biological effect of discovered mutations is often unknown (12). Moreover, recent studies suggest that tumor progression (9) and chemotherapy resistance (13) can occur in the absence of mutations. Therefore, the discovery and quantitative characterization of cancer cell fate-regulating networks is critically important to fully understand the mechanisms underlying cancer progression and to improve current treatment strategies.

Estrogen receptor alpha (ERα) is a major controller of normal mammary development and breast cancer progression (14). ERα-expressing cells differentiate to form a luminal epithelium coating the inner surface of mammary glands under normal circumstances. Breast cancers can be classified based on their ERα status as either ERα-positive or ERα-negative, the former being associated with better prognosis and response to hormone therapy treatment (15). Since no genetic mutations are known to underlie this classification (16), ERα status could be a purely phenotypic rather than genetically determined cell state. For example, an elevated ERα phenotype may arise as a result of gene regulation involving other TFs, such as GATA-binding protein 3 (GATA3).

GATA3 is a member of the GATA-binding TF family that contains zinc-finger motifs and promotes chromatin remodeling upon deoxyribonucleic acid (DNA) binding (17). Both ERα and GATA3 function as cell fate regulators in mammary gland development (18,19), promoting luminal cell differentiation (19,20). The expression levels of GATA3 in breast cancer patients are strongly correlated with ERα (21). Many studies have implicated GATA3 as a strong positive prognostic marker for breast cancer patients (22), with ERα-positive breast cancers having high GATA3 expression and well-differentiated cell morphology (23). In contrast, invasive ERα-negative cancers tend to have low GATA3 expression and poor cellular differentiation, with poor prognosis (22,24). The depletion of GATA3 in ERα-positive cell lines causes loss of ERα status and drives the cells to acquire metastatic characteristics (25,26). Concordantly, ectopic expression of GATA3 in basal-like breast cancer cell lines suppresses their metastatic potential and alters the tumor microenvironment (27). Likewise, GATA3 reconstitution in transgenic animal models suppresses the dissemination of breast cancer (23).

**Figure 1.**
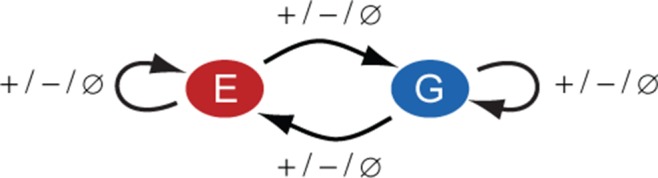
ERα–GATA3 network in breast cancer cells. The signs of autoregulatory and cross-regulatory connections in the ERα-GATA3 network are unknown and can be either positive (+), negative (−) or null ($\emptyset$).

The association of GATA3 expression levels with ERα-positive breast cancer hallmarks may be a consequence of transcriptional regulatory connections between the two TFs. Indeed, previous molecular-level studies indicated that ERα and GATA3 constitute a transcriptional regulatory network in ERα-positive breast cancer cell lines (28,29). These studies suggested that ERα and GATA3 autoregulate their own production and mutually regulate each other's expression by binding to relevant promoter and enhancer regions. However, many important details of this transcriptional regulatory network, such as the strength or sign of each regulatory connection remain unclear (Figure 1). This partly stems from the lack of biochemical information regarding TF binding to various DNA sites, interactions with co-regulators and enhancer activity (30,31) and partly from the lack of hypothesis-generating quantitative models that could be tested on experimental data. As a result, the functional significance of the correlation between ERα and GATA3 expression in breast cancer cell lines or in normal mammary gland development remains unclear.

Here, we synergistically combine mathematical modeling and perturbation experiments to unravel the transcriptional regulatory connections in the ERα–GATA3 network in ERα-positive breast cancer cell lines. Through cell population-average, single-cell and single-nucleus measurements we determine the sign of the cross-regulatory connections between ERα and GATA3. Subsequently, we use the mathematical model to ascertain the sign of autoregulation and to quantify the strength of various regulatory connections in the network. Finally, we show how eliminating either of the cross-regulatory connections elevates the noise of ERα or GATA3, indicating the physiological role of negative feedback in this developmental regulatory module.

## MATERIALS AND METHODS

### Cell culture

T47D and MCF7 human breast cancer cell lines were cultured at 37°C and 5% CO_2_ in DMEM (Cellgro) with phenol red, supplemented with 5% fetal bovine serum (FBS), 2 mM l-glutamine and 100 units/ml penicillin–streptomycin. Alternatively, cells were prepared in antibiotics-free and phenol red-free DMEM medium (Cellgro) supplemented with 5% charcoal dextran-stripped FBS 24 h before siRNA transfection. For hormone treatments, cells were grown for 48 h in phenol red-free DMEM supplemented with 5% dextran–charcoal treated FBS before treatment. Fulvestrant (ICI 182,780 or ICI) was added at the final concentration of 10 nM. The ethanol (EtOH) vehicle was used for control.

### siRNA transfection

Cells were prepared in a 60 mm dish for transfection for 24 h and grown in DMEM supplemented with 5% FBS without antibiotics. 60 nM of each siRNA duplex was transfected using oligofectamin RNAi max transfection reagent (Invitrogen) in Opti-MEM (Invitrogen) for 48–96 h. We used ON-TARGETplus SMARTpool siRNA sequences for human ERα (Dharmacon) or 5′-AGGCUCAUUCCAGCCACAGTT-3′ (28). The target sequence of GATA3 was 5′-AACAUCGACGGUCAAGGCAAC-3′ (28). Luciferase target sequence was used as non-specific control siRNA.

### Measurement of primary transcript levels

3′-UTR specific GATA3 siRNAs (SASI_Hs01_00153939 and SASI_Hs01_00153940) were purchased from Sigma Aldrich. After 48 h siRNA tansfection, the cells were transfected with GATA3 cDNA-containing plasmid for 48 h. Total ribonucleicacid (RNA) samples were collected and complementary DNA (cDNA) was generated using random primers. Exon–intron splicing boundary primer pairs that amplify only the primary transcript sequences (unspliced pre-mRNAs) were designed (Supplementary Table S2) and qRT-PCR was performed using SYBR Green (Applied Biosystems, API). Actin was used for internal control.

### Antibodies

The following antibodies were used for western blotting: mouse monoclonal anti-ERα (clone 6F11) was from Lab Vision Corp. Rabbit polyclonal anti-ERα (Clone 60c) for immunofluorescence and flow cytometry was from Millipore. Mouse monoclonal anti-GATA3 (HG3–31, sc-268) was purchased from Santa Cruz Biotechnology. Mouse monoclonal anti-actin (A5441) was from Sigma. Mouse monoclonal anti-vinculin (VG6110) was from Biomol.

### Reverse-phase protein array (RPPA)

Protein samples were prepared from T47D or MCF7 cell lines with siRNA for ERα or GATA3, respectively and RPPA was performed and validated (32). Briefly, in this analysis, the validated primary antibodies were probed and signal intensities were quantified by Microvigene software (VigeneTech Inc., Carlisle, MA). Average linkage hierarchical clustering was performed using Cluster 3.0 software and the colormap of protein expression levels was displayed by TreeView (http://rana.lbl.gov/EisenSoftware.htm).

### RNA isolation and quantitative real-time PCR (Polymerase Chain Reaction)

Total RNA was extracted from cells following the manufacturer's instructions (RNeasy mini kit, Qiagen). To measure ERα and GATA3 mRNA levels, real-time PCR was performed using the TaqMan Gene Expression Probes (Applied Biosystems, API) for each gene and Ambion one-step qRT-PCR kit with ABI sequence detection system (Applied Biosystems Instruments, API PRISM 7900HT). Standard qRT-PCR cycling conditions were used: 48°C for 30 min for reverse-transcription. 95°C for 10 min for initiation, followed by 40 cycles of 95°C for 15 s and 60°C for 60 s. Relative mRNA levels were calculated using the threshold cycle (CT). Cyclophilin A (PPIA) mRNA was used as the internal control.

### Chromatin immunoprecipitation (ChIP) assays

T47D cells were grown in 150 mm tissue culture plates and fixed with 1% formaldehyde for 10 min at room temperature. After fixation, chromatin samples were obtained using the enzymatic shearing method (ChIP-IT Express, Active Motif). Samples were immunoprecipitated with 2 μg of antibody against GATA3 (HG3-31, Santa Cruz). Mouse IgG (Active Motif) was used as negative control. Primer sets for ChIP-qPCR were designed as described in (28) and are listed in Supplementary Table S3. Real-time qPCR was performed using SYBR Green (Applied Biosystems, API) to assess DNA-binding fold changes.

### Flow cytometry

Cells were trypsinized and collected after 48 h siRNA transfection. For intensity measurement, cells were fixed with 90% methanol and analyzed using two-dimensional flow cytometry with ERα (Clone 60 c, Millipore 04-820) and GATA3 (HG3-31, Santa Cruz Biotechnology sc-268) antibodies. Alexa 488 (anti-mouse, green) and Alexa 647 (anti-rabbit, red) secondary antibodies were used. For each sample, ∼10 000 single cells were measured using BD FACScalibur (BD Bioscience). Flowjo (Tree Star) was used for analysis.

### Immunofluorescence

Cells were grown on poly-d-lysine coated coverslips and fixed with 4% paraformaldehyde (ultrapure, Electron Microscopy Sciences) for 30 min. After quenching with 100 mM ammonium chloride, cells were permeabilized with 0.1% Triton X-100 for 30 min and blocked with 4% milk for 1 h. Primary antibodies were incubated overnight at 4°C in 4% milk. Subsequently, cells were washed and incubated with secondary antibodies for 1 h at room temperature. After washing three times, cells were fixed again with 4% paraformaldehyde and quenched with 100 mM ammonium chloride. Cells were washed with TBS (Tris-buffered saline) and stained with DAPI (4',6-diamidino-2-phenylindole, 10 μg/ml) for visualizing their nucleus.

### Microscopy and image analysis

A DeltaVision Core fluorescence microscopy platform (Applied Precision) built upon an inverted microscope (1x70 Olympus) using 60x NA 0.4 oil objective was used to obtain constrained iterative deconvolved high resolution images. The Pipeline Pilot (version 7.5) software platform (Accelrys) equipped with the Advanced Imaging toolbox was used for image analysis (33). Using a custom automated image-analysis workflow, the background signal in each channel was subtracted and nuclear masks were generated using watershed masked clustering and non-linear least squares algorithms. Nuclear circularity, nuclear area and normal DNA contents (between 2C and 4C) were used to select nuclei for evaluation of pixel intensity values that were subsequently normalized by the area of each nucleus. ImageJ (NIH) was used for protein level quantification after western blotting.

### Mathematical model

We built an ordinary differential equation based phenomenological model for the ERα–GATA3 network. In this model, the network's autoregulatory and cross-regulatory connections were modeled using Hill functions. We assumed that autoregulatory and cross-regulatory connections combine together in a multiplicative fashion to regulate protein production. This is analogous to the multiplicative coupling of feedback loops (34). We also assumed first-order degradation for proteins, also incorporating dilution due to cell growth. The aforementioned assumptions resulted in the following differential equations that describe protein dynamics:

(1) \begin{equation*} \frac{{dE}}{{dt}} = b_e \frac{{\left( {1 + f_1 \frac{{E^{n_1 } }}{{K_1^{n_1 } }}} \right)}}{{\left( {1 + \frac{{E^{n_1 } }}{{K_1^{n_1 } }}} \right)}}\frac{{\left( {1 + f_2 \frac{{G^{n_2 } }}{{K_2^{n_2 } }}} \right)}}{{\left( {1 + \frac{{G^{n_2 } }}{{K_2^{n_2 } }}} \right)}} - k_e E
\end{equation*}

(2) \begin{equation*} \frac{{dG}}{{dt}} = b_g \frac{{\left( {1 + f_3 \frac{{E^{n_3 } }}{{K_3^{n_3 } }}} \right)}}{{\left( {1 + \frac{{E^{n_3 } }}{{K_3^{n_3 } }}} \right)}}\frac{{\left( {1 + f_4 \frac{{G^{n_4 } }}{{K_4^{n_4 } }}} \right)}}{{\left( {1 + \frac{{G^{n_4 } }}{{K_4^{n_4 } }}} \right)}} - k_g G
\end{equation*}

where *E* and *G* represent the concentrations of ERα and GATA3; $b_e$ and $b_g$ are the basal synthesis rates and $k_e$ and $k_g$ are the degradation rates of ERα and GATA3; $f_j$, $K_j$ and $n_j$ where $j \in \{ 1,2,3,4\}$ together describe the Hill function $F_j (X) = \frac{{\left( {1 + f_j \frac{{X^{n_j } }}{{K_j^{n_j } }}} \right)}}{{\left( {1 + \frac{{X^{n_j } }}{{K_j^{n_j } }}} \right)}}$, $X \in \{ E,G\}$. $f_j$ is the fold change in Hill function, *K_j_* is the protein concentration at which the function is half-maximally saturated and *n_j_* is the effective cooperativity.

To reduce the number of free parameters in the system we normalized the concentrations *E* and *G* with their respective normal (i.e. in the absence of any perturbation) cellular concentrations *E*_0_ and *G*_0_ to obtain normalized concentrations *e* and *g*. This gave rise to the following equations with dimensionless parameters apart from *f_j_* and *n_j_*:

\begin{eqnarray*} &&\tilde b_e = \frac{{b_e }}{{E_0 }},\quad \tilde b_g = \frac{{b_g }}{{G_0 }}, \\ &&\tilde K_1 = \frac{{K_1 }}{{E_0 }},\quad \tilde K_2 = \frac{{K_2 }}{{G_0 }},\quad \tilde K_3 = \frac{{K_3 }}{{E_0 }}\,\quad {\rm and}\,\quad \tilde K_4 = \frac{{K_4 }}{{G_0 }}.\end{eqnarray*}

As a result of the normalization all parameters in the above equations became dimensionless, except the protein degradation rates. The normalized concentrations and dimensionless parameters were used in all the simulations. Also, hereafter we drop the ∼ in the dimensionless parameters for sake of simplicity. Note that $F_1 (e)$ and $F_4 (g)$ represent the autoregulatory, whereas $F_2 (g)$ and $F_3 (e)$ represent the cross-regulatory connections in the network. Furthermore, *f_j_* < 1 and *f_j_* > 1 respectively depict negative and positive regulation, whereas *f_j_* = 1 depicts no regulation.

## RESULTS

### Cross-regulation between ERα and GATA3 results in a negative feedback

**Figure 2.**
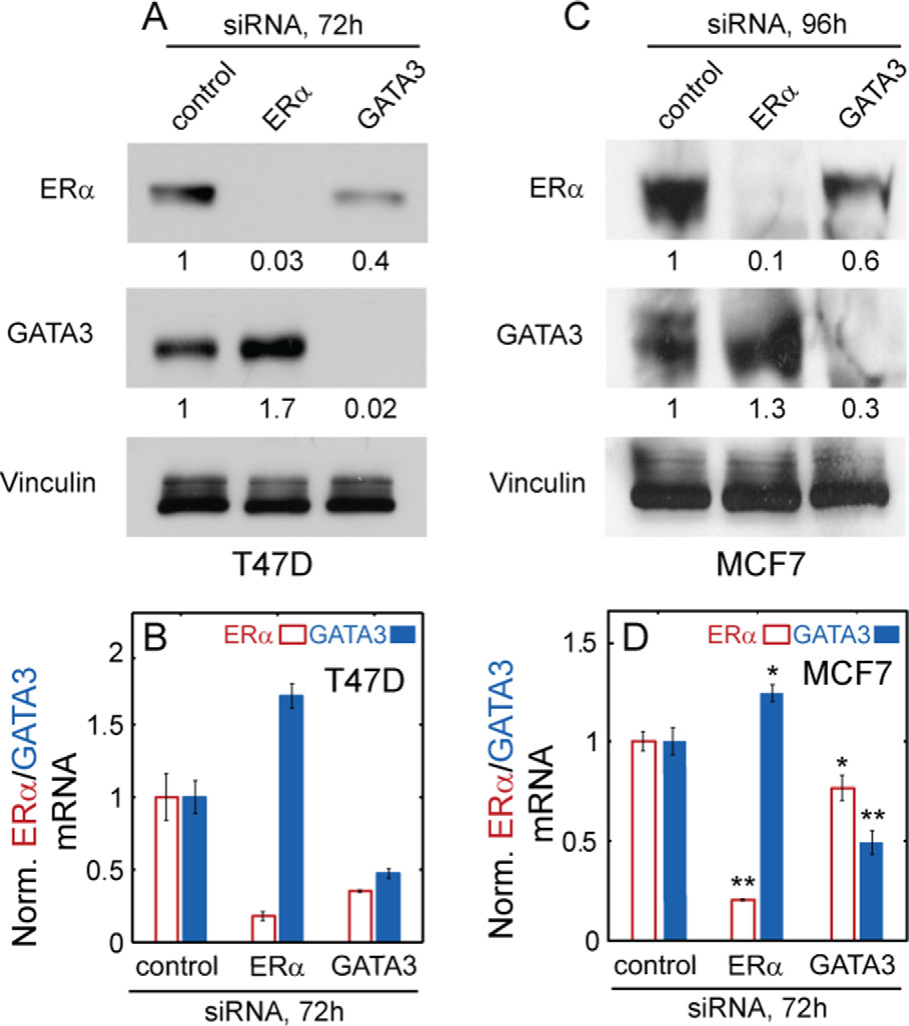
ERα and GATA3 asymmetrically regulate each other in ERα-positive breast cancer. **(A**) western blots depicting ERα and GATA3 protein levels in T47D cells that were transfected with control, ERα or GATA3 siRNA. Vinculin was used as the loading control. (**B)** qRT-PCR data depicting ERα and GATA3 mRNA levels in T47D cells after siRNA transfection. Error bars represent standard error (*n* = 3). (**C** and **D)** western blots and qRT-PCR data (*n* = 3) for MCF-7 cells after siRNA transfection. Band signals were quantitated and indicated in numbers (normalized by control). Student's *t*-test was used for statistical analysis. *P < 0.05 or **P < 0.01 versus control, respectively.

To determine the signs of cross-regulatory connections between ERα and GATA3, we used siRNA to decrease either ERα or GATA3 protein synthesis and then examined the change in ERα and GATA3 protein levels in the ERα–positive T47D breast cancer cell line. We observed, in accordance with a previous study (28), that GATA3 depletion caused a reduction in ERα protein levels measured by western blotting (a cell population-average measurement) (Figure 2A). In contrast, ERα-depleted cells showed a small, but significant increase in GATA3 protein levels (Figure 2A). Taken together, these observations indicate that GATA3 positively regulates ERα, whereas ERα negatively regulates GATA3, thereby constituting an overall negative feedback loop. We further confirmed these results by RPPA technology (Supplementary Figures S1A, S11C–D) to exclude the possibility of non-specific binding of antibodies in western blotting.

To confirm that these effects occurred through transcriptional regulation, we measured ERα and GATA3 mRNA levels through real-time qPCR (a cell population-average measurement) following either ERα or GATA3 siRNA treatment. In accordance with western blots, we found that ERα mRNA levels decreased due to GATA3 depletion, whereas GATA3 mRNA levels increased in ERα siRNA treated cells (Figure 2B). Conversely, ERα overexpression significantly repressed GATA3 mRNA and protein levels (Supplementary Figure S2A–C). Surprisingly, GATA3 overexpression did not have a significant effect on ERα expression (Supplementary Figure S2D–F**)**, possibly indicating that GATA3 may be present at saturating levels from the perspective of ERα regulation.

To test how the cross-regulation between ERα and GATA3 manifests at the single cell level, we used flow cytometry (following immunofluorescent labeling) to examine the change in the distributions of ERα and GATA3 protein levels after siRNA perturbation (Supplementary Figure S3A). In agreement with cell population-average measurements, ERα protein levels uniformly decreased, lowering the fluorescence mean by ∼50% in GATA3-depleted cells. Likewise, GATA3 protein levels slightly increased (∼15%) after ERα siRNA treatment. In addition, we used fluorescence microscopy to visualize ERα and GATA3 in the nuclei of T47D cells (Supplementary Figure S3B), and studied the effect of siRNA perturbations on the joint ERα–GATA3 probability distribution estimated from single-nucleus fluorescence intensities. In the GATA3 depleted samples the joint probability shifted toward low ERα/low GATA3 (Supplementary Figure S3C). On the other hand, depletion of ERα shifted the joint probability toward low ERα/high GATA3 (Supplementary Figure S3C). Besides being consistent with the cell population-average measurements, these shifts of ERα and GATA3 distributions suggest that the ERα–GATA3 network responds to perturbations in a uniform manner, maintaining a relatively low level of noise.

To determine whether the type of regulation is preserved in other cell lines, we performed similar experiments in another ERα-positive breast cancer cell line, MCF7. Protein and mRNA (Figure 2C and D and Supplementary Figure S1B) measurements in this cell line also showed ERα downregulation following GATA3 depletion and GATA3 upregulation following ERα depletion, indicating an overall negative feedback. However, in MCF7 cells the decrease in ERα following GATA3 siRNA treatment for 72 h was marginal in comparison to the effect observed in T47D (Supplementary Figure S4A). We speculated that this was due to the much slower degradation of ERα (half-life >8 h, Supplementary Figure S4B**)** in MCF7 cells, implying that 72 h transfection with GATA3 siRNA was insufficient for MCF7 cells to reach steady-state. Consequently, we incubated cells with siRNA for 96 h and finally observed ERα downregulation to a level similar as in T47D (Figure 2D). Such differences between cell lines may arise due to cell-type specific gene expression caused by chromatin remodeling, promoter region organization or mutation of regulatory genes (35–37). In summary, multiple lines of evidence indicate that GATA3 positively regulates ERα, whereas ERα exerts weak negative regulation on GATA3, resulting in an overall negative feedback in ERα-positive breast cancer cell lines.

### Mathematical modeling and time course experiments reveal that GATA3 positively regulates itself

While experimental examination of ERα and GATA3 levels after overexpression and depletion of each TF could suggest the signs (activating or repressing) of cross-regulatory connections, it cannot reveal their strengths. In addition, depletion by siRNA is too slow to separate cross-regulatory connections from autoregulatory effects. Therefore, to uncover the strengths and signs of all regulatory connections, we developed a mathematical model that could reveal these missing details when fit to experimental data.

**Figure 3.**
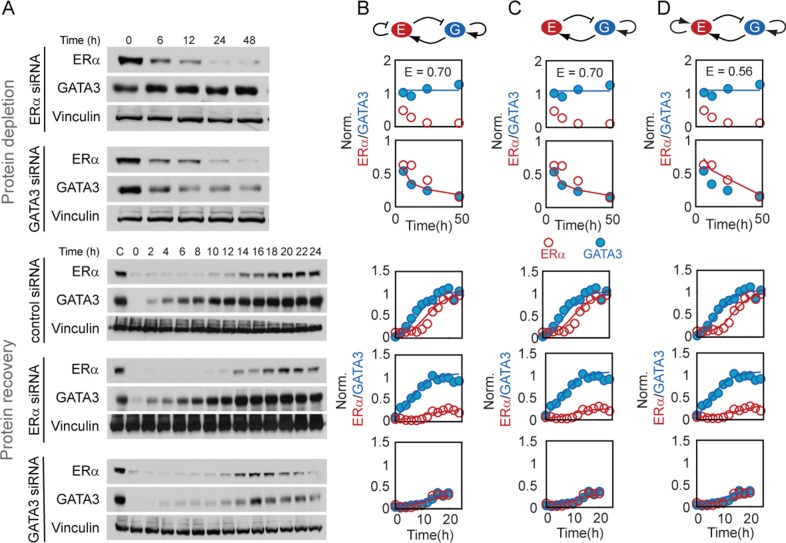
GATA3 positively regulates its own expression. (**A**) Protein depletion and protein recovery time courses monitored by western blotting (representative blot, *n* = 3 replicates). (B and D) Best model fits for topologies with negative (**B**), positive (**D**) or without (**C**) ERα autoregulation. Complete topologies with GATA3 autoregulation and cross-regulations are shown at the top of each column. Regular and blunt arrowheads depict positive and negative regulation. The open and solid circles in each panel indicate experimentally-measured ERα and GATA3 concentrations, obtained from panel (A) by western blot quantification. The continuous lines indicate model fits for protein depletion (top half) and recovery (bottom half) experiments. *E* is the error value of the fit. Model parameters for the three topologies in panels B–D are provided in Table S1.

The mathematical model consisted of two ordinary differential equations describing the dynamics of ERα and GATA3 proteins (see 'Materials and Methods' section for details). Regulatory connections between ERα and GATA3 could be positive, negative or absent (Figure 1) and were modeled by the range of parameter *f_j_*, quantifying the fold-change of the protein synthesis rates due to each interaction. For example, ERα may positively (*f*_1_ > 1) or negatively (*f*_1_ < 1) regulate its own expression or may not regulate (*f*_1_ = 1) itself at all. Since the ERα–GATA3 network contains two potential autoregulatory and two cross-regulatory connections, there are 3^4^ or 81 possible regulatory scenarios. To test that our optimization set-up was functioning correctly, we fit the model to data from ERα and GATA3 protein depletion experiments (Figure 3A, top panels) in which cells were incubated with siRNA. The fits (Supplementary Figures S5 and S6) confirmed the experimental findings that ERα negatively regulates GATA3, whereas GATA3 positively regulates ERα (Figure 2).

Autoregulation usually has a stronger effect on time courses compared to steady states (38). Therefore, to uncover the signs of autoregulatory connections we collected time-course data from protein recovery experiments consisting of three steps. First, we specifically depleted ERα or GATA3 by incubating the cells with the respective siRNA for the entire duration of the experiment. Second, after 48 h of incubation with siRNA we treated the cells with cycloheximide for 15 h to stop protein synthesis and thereby abate ERα and GATA3 protein expression. Third, we removed cycloheximide and monitored the recovery of protein levels through western blotting (Figure 3A, bottom panels). In these experiments, ERα protein recovery during ERα depletion is mainly governed by GATA3 regulation, whereas during GATA3 depletion it is mainly governed by ERα autoregulation. Likewise, GATA3 protein recovery in the presence of these siRNAs should be dominantly governed by either GATA3 autoregulation or ERα regulation. In contrast, protein recovery in the presence of control siRNA should have contributions from both autoregulatory and cross-regulatory connections.

To determine the relative contributions and signs of autoregulatory and cross-regulatory connections, we used protein recovery data for model fitting. We fixed the signs of cross-regulation between ERα and GATA3 as determined from experiments (Figure 2). As each of the two autoregulatory interactions could be either positive, negative or null, we independently fit 3^2^ or nine different model topologies to the data from protein recovery and siRNA transfection experiments. We found that only three topologies in which GATA3 positively regulated its own production could capture both datasets (Figure 3B–D), strongly supporting positive GATA3 autoregulation. ChIP assays confirmed that GATA3 was recruited to its own promoter or enhancer regions during protein recovery (Supplementary Figure S7). Further, we inhibited endogenous GATA3 mRNA by using 3′-UTR targeting siRNA and then rescued its functionality by protein overexpression to find that siRNA-immune exogenous GATA3 significantly increased its own primary transcript levels (Supplementary Figure S8). Taken together, these experiments provide firm support for GATA3 autoregulation.

Among the three topologies with positive GATA3 autoregulation, the one with positive ERα autoregulation (Figure 3D) had the minimum error. However, the error associated with the other two topologies was not significantly larger and given the uncertainty in experimental measurements we could not exclude them from further analysis. In summary, the modeling approach combined with the experimental data revealed the sign of GATA3 autoregulation which could not be extracted from a purely experimental analysis.

### ERα does not regulate its own production in the absence of estradiol

**Figure 4.**
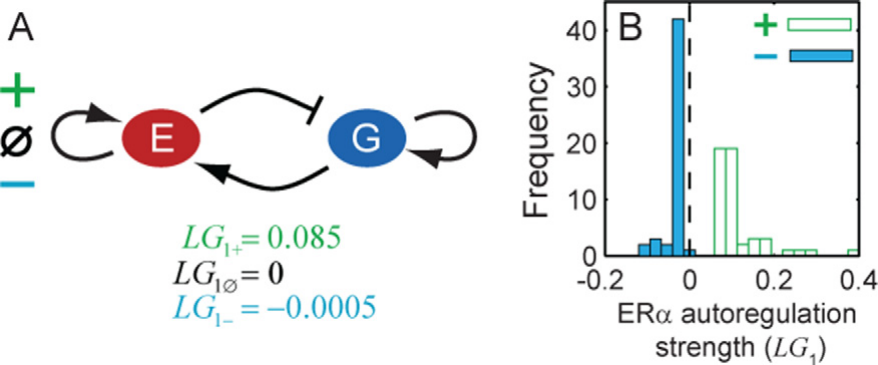
Model fitting constrains the topology with ERα negative autoregulation such that its strength becomes negligible **(A)** Strength of ERα autoregulation in the best fit case for the three possible autoregulation topologies—negative, null and positive. (**B)** Frequency histograms of ERα autoregulation strengths for topologies with negative (solid) and positive (open) autoregulation.

Model fitting could not uncover the sign of ERα autoregulation because we obtained three topologies with different signs of ERα autoregulation that could fit the protein recovery experimental data (Figure 3B–D). So we asked, is the topology with positive or negative ERα autoregulation significantly different from the one with no autoregulation? To answer this, we compared the strength of ERα autoregulation, measured as the logarithmic gain (${\rm LG}_1 = \frac{{d\log F_1 }}{{d\log e}}$ at *e* = 1) in these three topologies for the parameter sets resulting in best fits. We found that ${\rm LG}_1^ - \approx {\rm LG}_1^\emptyset {<} {\rm LG}_1^ +$ where $ - ,\,\,\emptyset \,\,{\rm and}\,\, +$ represent negative, null and positive ERα autoregulation (Figure 4A). This suggested that the best-fit model with negative ERα autoregulation topology is not different from the one with no autoregulation. In other words, our data constrained the negative autoregulation of ERα to be very weak. To further confirm this hypothesis, we extended this analysis in two ways. First, we examined autoregulation strengths in the 50 best-fit parameter sets for topologies with positive and negative ERα autoregulation. The frequency (Figure 4B) and cumulative probability (Supplementary Figure S9A) distributions of regulatory strengths for negative ERα autoregulation peaked sharply near zero, as opposed to the strictly positive range obtained for positive ERα autoregulation. Second, we computed the percentage change in error values after eliminating ERα autoregulation in the 50 best-fit parameter sets for the above two topologies. We observed that the change was ∼200% for the positive ERα autoregulation topology, whereas the change was negligible (<0.05)% for the negative ERα autoregulation topology (Supplementary Figure S9B and C). These additional analyses reinforced the observation that the negative ERα autoregulation topology fits the data only when the autoregulation is practically negligible. Consequently, we were left with only two feasible topologies, namely no autoregulation and positive ERα autoregulation.

**Figure 5.**
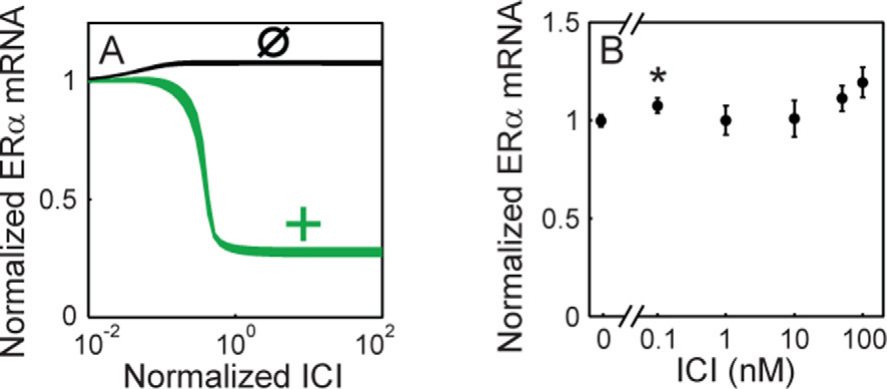
ERα is not autoregulated. **(A)** Computationally generated dose–response curves depicting the change in steady-state ERα mRNA levels with increasing concentrations of ICI for topologies with positive (dark gray) and without (black) autoregulation. For each topology the dose–response curves for the best 50 parameter sets are plotted. (**B)** ERα mRNA levels in the T47D cell line after 24 h incubation with different concentrations of ICI (*n* = 3). Note that only the 0.1 nM measurement is significantly different (*P* < 0.05 by Student's *t*-test) from the control sample (0 nM).

To distinguish between these two remaining topologies, we decided to use the model to design another perturbation that could reveal the sign of ERα autoregulation. The chemical compound ICI182,780 (ICI hereafter) is known to quickly sequester ERα from the nucleus and induce its depletion independently of transcription (39,40). Therefore, we used our model to examine the change in steady state ERα mRNA levels as a function of ICI. We observed that the dose–response curves for the 50 best-fit parameter sets were qualitatively different for the two topologies—monotonically decreasing for positive ERα autoregulation and monotonically increasing for no ERα autoregulation (Figure 5A). At the same time the dose–response curves for GATA3 mRNA, ERα protein and GATA3 protein were qualitatively similar for the two topologies (Supplementary Figure S10). Therefore, the trend of ICI dose–response should distinguish between the positive and null ERα autoregulatory topologies. Encouraged by this prediction, we measured ERα mRNA levels experimentally after incubating the cells with different doses of ICI for 24 h to allow the system to reach steady state. ERα mRNA levels increased slightly with increasing doses of ICI, which was consistent with the simulated dose–response curves for no ERα autoregulation. Thus, this experiment suggested by the model indicated that ERα does not regulate its own production (Figure 5B).

To further confirm the lack of ERα autoregulation, we took advantage of the quick sequestration and depletion of ERα protein following ICI treatment. If autoregulation were present, ERα mRNA levels should change immediately after ERα protein depletion. However, western blotting and real-time qPCR (Figure 5B and Supplementary Figure S11A**)** indicated that ERα mRNA levels were not significantly affected immediately after ICI treatment, while ERα protein levels quickly dropped, supporting the lack of ERα autoregulation (Figure 5B). At the same time, GATA3 mRNA levels increased significantly upon ICI treatment, confirming that ERα represses GATA3 (Supplementary Figure S11B).

### Negative feedback reduces noise in the ERα–GATA3 network

Having uncovered the regulatory connections in the ERα–GATA3 network, we asked whether this particular architecture had any features that would make it physiologically optimal. The network was monostable for the best model parameter sets, arguing against a role for stochastic switching (41–43) or stable diversification (44–46). Considering that cells in the mammary gland are constantly exposed to fluctuations in growth factors and the estrogen hormone, in addition to intrinsic gene expression noise (47–50), the ERα–GATA3 network architecture may be suited to ensure robust cell state maintenance in a highly variable environment. While this purpose is well-justified in normal circumstances, it may also be preserved in disease conditions, such as ERα-positive breast cancer.

**Figure 6.**
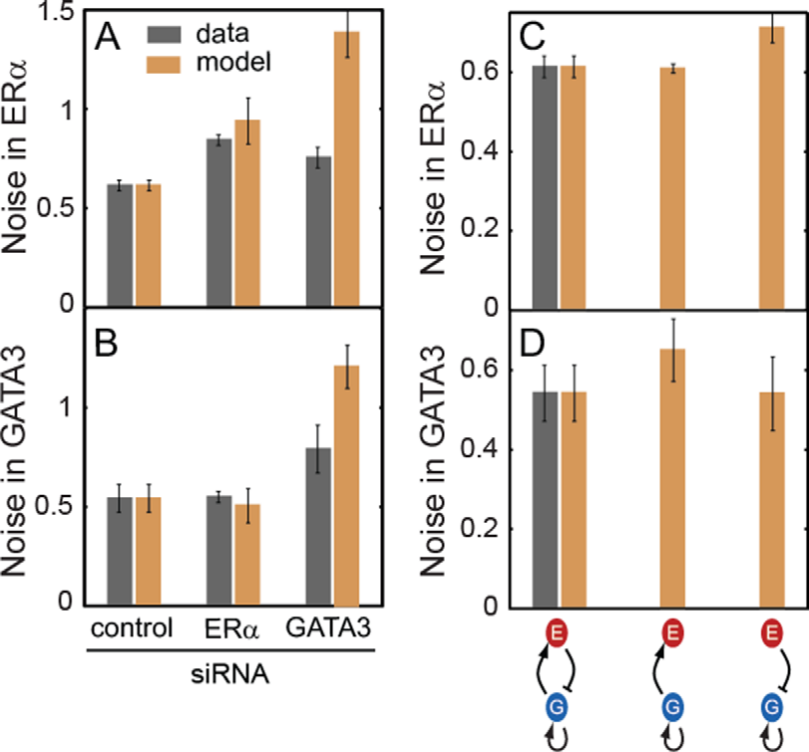
Model with only intrinsic noise predicts that negative feedback suppresses noise in ERα and GATA3 levels. **(A** and **B)** Noise in ERα and GATA3 levels in T47D cells after transfection with control, ERα or GATA3 siRNA for 48 h (dark gray bars). In the model, noise was first matched to the levels observed experimentally with control siRNA and later the model was used to predict noise for ERα or GATA3 siRNA treatment (**C** and **D).** WT bars are the same as for control siRNA in panels A and B. The model predicts that either ERα or GATA3 noise levels increase in the mutants without negative feedback. Error bars represent standard error (*n* = 3).

To examine how network architecture affects its robustness to random intrinsic fluctuations (gene expression noise), we calculated the noise in ERα and GATA3 expression using the Linear Noise Approximation (51). Noise levels (CV = *σ*/*μ*) calculated for unperturbed (wild-type, WT), ERα-depleted and GATA3-depleted conditions (Figure 6A and B) were in good qualitative agreement with the values obtained by single-nucleus measurements in the same conditions (Supplementary Figure S3B), further confirming the validity of our model. Next, we calculated the noise in ERα and GATA3 expression after eliminating the negative feedback in the model by either breaking the ERα activation by GATA3 or GATA3 repression by ERα. To enable controlled comparison (52) we adjusted the basal production rate of ERα or GATA3 such that their means matched their levels in unperturbed conditions. Interestingly, the noise in either ERα or GATA3 (but not both) increased significantly above WT level in the absence of negative feedback (Figure 6C and D. Moreover, the noise-reducing effect of negative feedback was also observed in the case of extrinsic (parameter) noise (Supplementary Figure S12). The relatively low noise of ERα and GATA3 expression levels in single cell- and single nucleus-level measurements (Supplementary Figure S3) further corroborate these findings. The results for extrinsic noise were completely consistent with those obtained for intrinsic noise. Overall, the above analyses suggest that the ERα–GATA3 network architecture ensures cell state resilience to intrinsic and extrinsic fluctuations in the normal mammary gland, which is preserved in ERα-positive breast cancer cells.

## DISCUSSION

**Figure 7.**
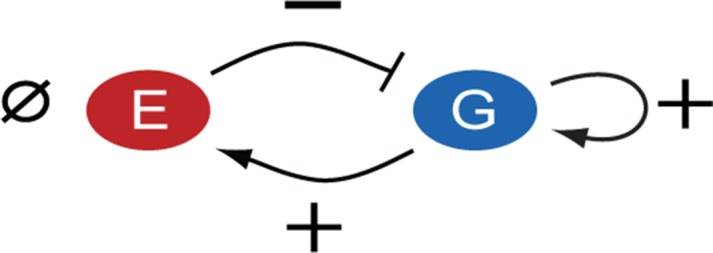
ERα−GATA3 network identified in breast cancer cells. The ERα–GATA3 network consists of an overall negative feedback loop and GATA3 positive autoregulation. The signs of regulatory interactions are indicated as positive (+), negative (−) or null ($\emptyset$).

Cancer arises from the disruption of normal tissue homeostasis, which relies on transcriptional and post-transcriptional control of genes responsible for proliferation, differentiation and apoptosis. Despite the ever-increasing list of genes implicated in cancer progression, we still lack a quantitative understanding of the regulatory networks that control many cancer-related genes. This deficiency hinders the mechanistic understanding of cancer progression and may be a roadblock to developing more efficient cancer treatments. For example, the transcriptional regulation of ERα, one of the most important genes in breast cancer is still not well understood. Although prior findings suggested that ERα and GATA3 regulate themselves and each other (28), the details of these regulatory connections were unknown. Here we characterized the regulatory connections between ERα and GATA3 in ERα-positive breast cancer cell lines. We found that the cross-regulation between ERα and GATA3 gives rise to a negative feedback loop and GATA3 positively regulates its own production, whereas ERα does not regulate itself (Figure 7).

Given the ERα-dependent classification of breast cancers into two major subtypes (ER-positive and ER-negative), we naïvely expected that a quantitative characterization of the ERα–GATA3 network would reveal a bistable switch (45,46,53–58). However, we found that the network is monostable in the ERα high/GATA3 high state for the best-fit parameter set. Nevertheless, we could reach the ERα low/GATA3 low state through a specific perturbation in ERα-positive breast cancer cell lines (Figure 3A top panel, compare protein levels at 0 and 48 h in the GATA3 depletion experiments). In this respect GATA3 siRNA mimics the effect of an unknown upstream factor that regulates the transition between the two ERα-expression states. Going forward it will be important to identify these unknown factors to better understand the origins of intra-tumor and inter-patient diversity in breast cancers. Alternatively, one can use the siRNA-dependent network perturbations employed here to investigate how different gene expression states affect the phenotypes of breast cancer cells.

Admittedly, ERα regulation is highly complex and involves other TFs besides GATA3. The effect of these TFs is incorporated implicitly in the current model and may need to be included explicitly in the future. For example, another TF, FOXA1 initiates ERα recruitment to promoter regions relevant to breast cancer (59) and mediates epigenetic control of cell-type specific gene expression (60). FOXA1 is co-expressed with ERα and GATA3 and appears to be related to the luminal subtype A in breast cancer. These three TFs bind to each other's promoter regions (61,62), and importantly the FOXA1-mediated DNA-binding capacity of ERα relates to breast cancer risk (63). Thus, FOXA1 plays a crucial role in determining breast cancer gene expression profiles (64), and including it in a similar analysis may further improve our understanding of the transcriptional regulation of ERα.

Apart from incorporating other regulators, an additional challenge is to investigate the response of the current or extended versions of the ERα regulatory network to its natural ligand estradiol. The transcriptional activity of ERα is enhanced in the presence of estradiol (65). The presence of ligand may strengthen negative ERα autoregulation, as opposed to relatively weak or non-existent autoregulation in the current analysis. This strong negative autoregulation could contribute to the decrease in ERα protein levels observed upon treatment with estradiol (67). Based on these observations we speculate that the negative feedback in the ERα–GATA3 network may become stronger in the presence of estradiol and ultimately may limit (66) the genome-wide ERα response to ligand.

While our experiments provided qualitative information about the cross-regulatory connections, the mathematical model was necessary for a quantitative understanding of the network. In fact, we were unable to unravel the autoregulatory connections solely through experiments. Only by iteratively utilizing the mathematical model to suggest new experiments and to interpret resulting data could these connections be quantitatively characterized beyond doubt. In the course of these interdisciplinary efforts, we have developed a novel double-perturbation method involving siRNA and cycloheximide and mathematical modeling to specifically characterize the autoregulatory connections in the network. This method can be easily applied to characterize autoregulatory connections in any two-component network as long as siRNAs or chemical compounds are available to specifically perturb network proteins. Potentially, the method can also be extended to study more complicated network topologies, but the combinatorial complexity in such cases may confound the analysis. Overall, our work exemplifies how network complexity (such as multiple feedback loops) can pose a challenge to experimentalists that can be resolved once mathematical modeling is applied to analyze systematic network perturbation data (68–70).

Finding network structures that result in a certain type of time-course data is an emerging challenge in systems biology. For example, the topologies capable of producing perfect adaptation have been identified by searching a large network space (71). Our work aligns well with such efforts, with the added benefit of matching specific experimental data. Our methods are generalizable and could be deployed to unravel and characterize other networks regulating development or other types of cancer, which is crucial for mechanistic, quantitative understanding and possibly future control of cancer and mammalian development.

## SUPPLEMENTARY DATA

Supplementary Data are available at NAR Online.

SUPPLEMENTARY DATA
