# Supplementary material for: Unraveling the regulatory connections between two controllers of breast cancer cell fate
Source: Nucleic Acids Res. 2014 May 3;42(11):6839–49. doi: 10.1093/nar/gku360 (PMC4066784; doi:10.1093/nar/gku360)
Supplement: SUPPLEMENTARY DATA [file supp_42_11_6839__index.html]

Unraveling the regulatory connections between two controllers of breast cancer cell fate — SUPPLEMENTARY DATA 

# Unraveling the regulatory connections between two controllers of breast cancer cell fate

## SUPPLEMENTARY DATA

**Files in this Data Supplement:**

- SUPPLEMENTARY DATA
