# Supplementary material for: Unraveling the regulatory connections between two controllers of breast cancer cell fate
Source: Nucleic Acids Res. 2014 May 3;42(11):6839–49. doi: 10.1093/nar/gku360 (PMC4066784; doi:10.1093/nar/gku360)
Supplement: SUPPLEMENTARY DATA [file supp_gku360_JinhoLee_NAR-00188-H-2014_SI_gku360.docx]

**Supplementary Data**

**Unraveling the regulatory connections between two controllers of breast cancer cell fate**

Jinho Lee^1,2†^, Abhinav Tiwari^2†^, Victor Shum^1,3^, Gordon B. Mills^1^, Michael A. Mancini^4^, Oleg A. Igoshin^2*^ and Gábor Balázsi^1*^

**Affiliations:**

^1^ Department of Systems Biology – Unit 950, The University of Texas MD Anderson Cancer Center, Houston, TX 77054, USA.

^2^ Department of Bioengineering, Rice University, Houston, TX 77005, USA.

^3^ Department of Physics, University of Houston, Houston, TX 77004, USA.

^4^ Department of Molecular and Cellular Biology, Baylor College of Medicine, Houston, TX 77030, USA.

*To whom correspondence should be addressed:

Tel: +1-713-834-6157; Fax: +1-713-563-4235

E-mail:

[gbalazsi@mdanderson.org](mailto:gbalazsi@mdanderson.org)

[gabor@laufercenter.org](mailto:gabor@laufercenter.org)

or

E-mail: [igoshin@rice.](mailto:igoshin@rice.)edu; Tel: +1- 713-348-5502; Fax: +1- 713-348-5877

^†^ These authors contributed equally to this work.

**Contents**

Supplementary Text

Table S1. Normalized model parameters for the three network topologies in Figure 3B-D

Table S2. Primer sets used for GATA3 primary transcript level measurements

Table S3. Primer sets used for ChIP-qPCR

Figure S1. RPPA analysis confirms asymmetric regulation between ERα and GATA3

Figure S2. Overexpression analysis confirms asymmetric mutual regulation between ERα and GATA3

Figure S3. Single cell- and single nucleus-level measurements confirm the asymmetry of regulatory connections between ERα and GATA3

Figure S4. ERα degradation rate is slower in MCF-7 cells compared to T47D cells

Figure S5. Model fits for ERα  siRNA transfection experiments show that ERα negatively regulates GATA3

Figure S6. Model fits for GATA3 siRNA transfection experiments show that GATA3 positively regulates ERα

Figure S7. GATA3 binds to promoter and enhancer regions of ERα and itself to regulate protein synthesis during recovery

Figure S8. GATA3 is under a positive autoregulation.

Figure S9. The topology with ERα negative autoregulation is equivalent to no autoregulation

Figure S10. Dose-response curves for protein levels are qualitatively similar for the topologies without and with positive ERα autoregulation

Figure S11. ICI-mediated perturbation confirms that ERα negatively regulates GATA3

Figure S12. The model with only extrinsic noise predicts that negative feedback suppresses noise in ERα and GATA3 levels

Figure S13. Estimating ERα  and GATA3 protein degradation rates

References

**Supplementary Text**

**Modeling the effect of ICI**

ICI is a chemical compound that sequesters ERα from nucleus and induces its degradation in the cytoplasm (1, 2). ERα bound to ICI cannot bind to DNA and hence is transcriptionally inactive (2). To incorporate the two effects of ICI we included the following reactions:

where $k_{b}$ and $k_{d}$ are the rates of binding and dissociation of ICI, $\lambda$ is the ratio of the degradation rates of ERα bound to ICI and free ERα. In our simulations we set $\lambda=3$ based on the data from pulse-chain experiments which measured protein half-life in the presence and absence of ICI (1).

The inclusion of ICI resulted in the following differential equations for the two ERα species:

$$\frac{de}{dt}=b_{e}\frac{\left(1+f_{1}\frac{e^{n_{1}}}{K_{1}^{n_{1}}}\right)}{\left(1+\frac{e^{n_{1}}}{K_{1}^{n_{1}}}\right)}\frac{\left(1+f_{2}\frac{g^{n_{2}}}{K_{2}^{n_{2}}}\right)}{\left(1+\frac{g^{n_{2}}}{K_{2}^{n_{2}}}\right)}-k_{b}ei+k_{d}(e\text{-}i)-k_{e}e$$

$$\frac{d(e\text{-}i)}{dt}=k_{b}ei-k_{d}(e\text{-}i)-\lambda k_{e}(e\text{-}i)$$

where $e$ and $(e\text{-}i)$ represent the normalized concentrations of free ERα and ERα bound to ICI (both species are normalized by ERα concentration in unperturbed cells). $k_{b}$ and $k_{d}$ now represent dimensionless rate parameters which were set to 0.1 and 0.001 respectively. Note that the qualitative response observed does not depend on the exact values of these two parameters. The differential equation governing GATA3 dynamics (normalized version of equation ) is unchanged as only free ERα functions as a TF. In the simulations the amount of ICI in the system is tuned by varying the parameter $i$.

**Model with only intrinsic noise**

We used Linear Noise Approximation (3) to compute the intrinsic noise in ERα and GATA3 in the model. This approximation is employed to gain a qualitative understanding of noise in the network which is valid over the range of parameter values. In this method we used equations and and solved the fluctuation-dissipation matrix equation (**A**σ + σ**A**^T^ + **B =** 0) at steady state for covariance matrix σ (4). Here, **A** is the Jacobian of the system and **B** is the diffusion matrix. Using equations and ,

$A\text{ = }\left(\begin{matrix} b_{e}F_{2}\frac{dF_{1}}{dE}-k_{e} & b_{e}F_{1}\frac{dF_{2}}{dG} \\ b_{g}F_{4}\frac{dF_{3}}{dE} & b_{g}F_{3}\frac{dF_{4}}{dG}-k_{g} \end{matrix}\right)$ and $B\text{ = }\left(\begin{matrix} b_{e}F_{1}F_{2}+k_{e}E & 0 \\ 0 & b_{g}F_{3}F_{4}+k_{g}G \end{matrix}\right)$

The matrices are computed at steady-state i.e. $b_{e}F_{1}F_{2}=k_{e}E$ and $b_{g}F_{3}F_{4}=k_{g}G$ (from equations and in the main text). Using these relations the above matrices were simplified to:

$A \text{= }\left(\begin{matrix} k_{e}\frac{E}{F_{1}}\frac{dF_{1}}{dE}-k_{e} & b_{e}\frac{E}{F_{2}}\frac{dF_{2}}{dG} \\ k_{g}\frac{G}{F_{3}}\frac{dF_{3}}{dE} & k_{g}\frac{G}{F_{4}}\frac{dF_{4}}{dG}-k_{g} \end{matrix}\right)=\left(\begin{matrix} k_{e}(LG_{1}-1) & \rho k_{e}LG_{2} \\ \frac{k_{g}}{\rho}LG_{3} & k_{g}(LG_{4}-1) \end{matrix}\right)$ and $B\text{ = }\left(\begin{matrix} 2k_{e}E & 0 \\ 0 & 2k_{g}G \end{matrix}\right)$

where $LG_{j}=\frac{X}{F_{j}}\frac{dF_{j}}{X}, X\in\{E,G\}$ is the steady state logarithmic gain for the *j*^th^ regulatory connection, and $\rho=\frac{E}{G}$ is the ratio of steady-state concentrations of ERα and GATA3.

Next we replaced $LG_{j}$ by $H_{j}$ ($H_{1}=1-LG_{1}$, $H_{2}=-LG_{2}$, $H_{3}=-LG_{3}$, $H_{4}=1-LG_{4}$) and substituted the various matrices in the fluctuation-dissipation matrix equation to obtain:

$$\left(\begin{matrix} k_{e}H_{1} & \rho k_{e}H_{2} \\ \frac{k_{g}H_{3}}{\rho} & k_{g}H_{4} \end{matrix}\right)\left(\begin{matrix} \sigma_{EE} & \sigma_{EG} \\ \sigma_{GE} & \sigma_{GG} \end{matrix}\right)+\left(\begin{matrix} \sigma_{EE} & \sigma_{EG} \\ \sigma_{GE} & \sigma_{GG} \end{matrix}\right)\left(\begin{matrix} k_{e}H_{1} & \frac{k_{g}H_{3}}{\rho} \\ \rho k_{e}H_{2} & k_{g}H_{4} \end{matrix}\right)=\left(\begin{matrix} 2k_{e}E & 0 \\ 0 & 2k_{g}G \end{matrix}\right)$$

The above equation is solved for $\sigma_{EE}$ and $\sigma_{GG}$ which are then used to compute noise in ERα $(\eta_{E})$ and GATA3 $(\eta_{G})$:

$$\eta_{E}=\frac{\sqrt{\sigma_{EE}}}{E}=\sqrt{\frac{1}{H_{1}(H_{1}H_{4}-H_{2}H_{3})}\left[\frac{H_{1}H_{4}-\lambda_{e}H_{2}H_{3}}{E}+\frac{\lambda_{e}H_{2}^{2}}{G}\right]}$$

$$\eta_{G}=\frac{\sqrt{\sigma_{GG}}}{G}=\sqrt{\frac{1}{H_{4}(H_{1}H_{4}-H_{2}H_{3})}\left[\frac{H_{1}H_{4}-\lambda_{g}H_{2}H_{3}}{G}+\frac{\lambda_{g}H_{3}^{2}}{E}\right]}$$

where $\lambda_{e}=\frac{k_{e}H_{1}}{k_{e}H_{1}+k_{g}H_{4}}$ and $\lambda_{g}=1-\lambda_{e}$.

Finally, ERα and GATA3 concentrations in the above noise expressions were normalized as described earlier to arrive at the analytical expressions which were used for all calculations.

In the above expressions all quantities except $E_{0}$ and $G_{0}$ are determined by model parameters. Therefore, we fitted the above expressions to experimentally-determined CVs (CV = σ/µ, n = 3) for ERα and GATA3 in the control siRNA (or WT) case to obtain *E_0_* and *G_0_*. To predict noise in the presence of ERα or GATA3 siRNA we decreased the respective protein’s basal production rate such that the fold decrease in the mean protein level in the model matches that in the experimental data and re-computed $\eta_{e}$ and $\eta_{g}$. To predict noise in the negative feedback deletion mutants we first established controlled comparison by adjusting the basal production rate of ERα (in mut1) or GATA3 (in mut2) such that the mean protein level matches that in WT, and then re-computed $\eta_{e}$ and $\eta_{g}$.

**Model with only extrinsic noise**

We assumed that extrinsic noise primarily affects the processes of protein production and degradation. From a modeling perspective this amounts to variability in the corresponding reaction rates $b_{e}$, $b_{g}$, $k_{e}$ and $k_{g}$. We introduced this parameter variability in a population of cells as follows, for each cell:

i) draw independent random numbers $\xi_{1}$ and $\xi_{2}$ from two lognormal distributions with the same mean (=1), but different variances ($v_{1}$ and $v_{2}$),

ii) redefine $b_{i}^{cell}=b_{i}\xi_{1}$ and $k_{i}^{cell}=k_{i}\xi_{2}$, $i\in\{e,g\}$, where $b_{i}$ and $k_{i}$ are the parameter values for the best fit case,

iii) compute steady state normalized concentrations of ERα and GATA3 using equations (1) and (2),

iv) multiply the values obtained in iii) with $E_{0}$ and $G_{0}$ to convert them into non-normalized concentrations.

Thus, we obtained the distributions of ERα and GATA3 levels in the population of cells which were subsequently used to compute CVs. Note that the scaling of production and degradation rates in ii) biologically corresponds to the assumption that extrinsic noise results in global fluctuations in protein production and degradation in population of cells (ref 47 in main text). On the other hand, two independent random numbers used in i) signifies that fluctuations in production and degradation are uncorrelated.

Similarly to the case of intrinsic noise $E_{0}$ and $G_{0}$ are unknown in the above process and were estimated to be 30.05 and 71.34 respectively by fitting the means of distributions from the model to the experimentally-determined means in the control siRNA case. Moreover, $v_{1}$ and $v_{2}$ are also unknown in the above process and were estimated to be 0.37 and 0.29 respectively by fitting the CVs from simulations to experimentally-determined CVs in the control siRNA case. To predict noise in the presence of ERα or GATA3 siRNA and in the mutants we followed the same procedure as described in the section on intrinsic noise.

**Optimization**

All objective function minimizations were performed using the generic particle-swarm optimization (PSO) MATLAB function (5). The default options structure of *pso* was used for all optimizations with two modifications: the number of particles in the swarm and the maximum number of generations were set to 20 and 1000 respectively. We also included the option of a hybrid function in which *fmincon* continues the optimization locally after PSO is terminated. To fit a model topology to experimental data optimization was repeated at least 250 times so that the best 5 fits converge to the same minimum value of the objective function. In optimization the various dimensionless parameters in the model were selected from the bounds listed in the following table:

**Table 1: Parameter bounds for optimization**

| **Parameter** | **Lower bound** | **Upper bound** |
| --- | --- | --- |
| $f_{j} , j\in\{1, 2, 3, 4\}$ | 0.01 | 100 |
| $K_{j},j\in\{1, 2, 3, 4\}$ | 0.01 | 100 |
| $n_{j} ,j\in\{1, 2, 3, 4\}$ | 0.1 | 10 |
| $\alpha_{e}(\text{ERα} \text{siRNA})$ | 0.01 | 1 |
| $\alpha_{g}(\text{GATA3} \text{siRNA})$ | 0.01 | 1 |

**Fitting the data from protein recovery experiments**

The equations and objective functions used to fit the model to data from protein depletion experiments are described in Supplementary Material. Here we describe the process of fitting the model to data from protein recovery experiments. In this case the cross-regulation between ERα and GATA3 was fixed based on the experimental results (**Figure 2**), i.e. $f_{3}<1$ and $f_{2}>1$. In the presence of a siRNA, the respective mRNA is degraded and as a result the rate of protein production decreases. To account for this effect on system dynamics we introduced two parameters $\alpha_{e}$ and $\alpha_{g}$ which are multiplied to the basal synthesis rates of ERα and GATA3 in their differential equations. In the simulations for protein recovery experiments with i) ERα siRNA $\alpha_{e}<1, \alpha_{g}=1$, ii) GATA3 siRNA $\alpha_{e}=1, \alpha_{g}<1$ and iii) control siRNA $\alpha_{e}=\alpha_{g}=1$. The differential equations for the three types of siRNAs were solved using *ode23s* in MATLAB (The Mathworks Inc., Natick, MA), and the concentrations of ERα and GATA3 thus obtained were used to minimize the following objective function for $\vec{p}=\{f_{1},K_{1},n_{1},f_{2},K_{2},n_{2},f_{3},K_{3},n_{3},f_{4},K_{4},n_{4},\alpha_{e},\alpha_{g}\}$:

$O\left(\vec{p}\right)=\sqrt{O_{dyn}\left(\vec{p}\right)+O_{ss}\left(\vec{p}\right)}$ (S1)

where,

$O_{dyn}\left(\vec{p}\right)=\sum_{i} O_{dyn}^{i}\left(\vec{p}\right)$, *i* ϵ [control siRNA, ERα siRNA, GATA3 siRNA]

$O_{dyn}^{i}=\sum_{t} \left(e_{t}-e_{t}^{obs}\right)^{2}+\left(g_{t}-g_{t}^{obs}\right)^{2}$, *t* represents experimental time points

$\left(e_{t},g_{t}\right)$ and $\left(e_{t}^{obs},g_{t}^{obs}\right)$ are (ERα, GATA3) concentrations in simulations and experiments. , *j* ϵ [ERα siRNA, GATA3 siRNA]. $O_{ss}^{j}$ is defined in the next section.

As the two autoregulatory interactions $(f_{1}, f_{4})$ can be positive, negative or null, we minimize the objective function in (S1) for 3^2^ or 9 different topologies.

**Fitting the data from protein depletion experiments**

To test our optimization set-up we fit the model to data from protein depletion (via siRNA transfection) experiments (**Figure 3A, top**). In these experiments we incubated the cells with a fixed concentration of ERα or GATA3 siRNA for different durations (6h, 12h, 24h and 48h), and in the end measured protein concentrations using immunoblotting. siRNA functions by promoting the degradation of mRNA thereby depleting the protein concentrations. This is a complex process which involves delivery of siRNA to cells, cellular uptake of siRNA and its transportation to the nucleus. However, we do not aim to study the detailed system dynamics, but want to use our model to only explain the perturbation effects. Hence, while modeling the effect of a siRNA in the protein depletion experiments we ignored the differential equation that describes the dynamics of siRNA target protein. As a result, we ended up with only one differential equation for the non-siRNA target protein. Moreover, since the half-lives of ERα and GATA3 are short (< 3h, see Methods section in the main text for details) we assumed that this differential equation is at quasi-steady state at all experimental end points. Below we present the equations used to model ERα and GATA3 siRNA transfections:

ERα siRNA

At $t=0h$, before the transfection of siRNA the two proteins are at their normal cellular concentrations i.e. $e=g=1$. Substituting these in the differential equation for GATA3 (normalized version of equation (2) in the main text) at quasi-steady state, we obtained the following expression for basal synthesis rate of GATA3 $(b_{g})$:

$b_{g}=k_{g}\frac{\left(1+\frac{1}{K_{3}^{n_{3}}}\right)}{\left(1+f_{3}\frac{1}{K_{3}^{n_{3}}}\right)}\frac{\left(1+\frac{1}{K_{4}^{n_{4}}}\right)}{\left(1+f_{4}\frac{1}{K_{4}^{n_{4}}}\right)}$ (S2)

Now if $(e_{t},g_{t})$ be the concentrations of ERα and GATA3 at any experimental end point $t$ where $t\in\{6h, 12h, 24h, 48h\}$, then following the above steps:

$b_{g}\frac{\left(1+f_{3}\frac{e_{t}^{n_{3}}}{K_{3}^{n_{3}}}\right)}{\left(1+\frac{e_{t}^{n_{3}}}{K_{3}^{n_{3}}}\right)}\frac{\left(1+f_{4}\frac{g_{t}^{n_{4}}}{K_{4}^{n_{4}}}\right)}{\left(1+\frac{g_{t}^{n_{4}}}{K_{4}^{n_{4}}}\right)}=k_{g}g_{t}$ (S3)

Next, we substituted the expression for $b_{g}$ from equation (S2) in the above equation:

$\frac{1}{g_{t}}\frac{\left(1+f_{3}\frac{e_{t}^{n_{3}}}{K_{3}^{n_{3}}}\right)}{\left(1+\frac{e_{t}^{n_{3}}}{K_{3}^{n_{3}}}\right)}\frac{\left(1+f_{4}\frac{g_{t}^{n_{4}}}{K_{4}^{n_{4}}}\right)}{\left(1+\frac{g_{t}^{n_{4}}}{K_{4}^{n_{4}}}\right)}=\frac{\left(1+f_{3}\frac{1}{K_{3}^{n_{3}}}\right)}{\left(1+\frac{1}{K_{3}^{n_{3}}}\right)}\frac{\left(1+f_{4}\frac{1}{K_{4}^{n_{4}}}\right)}{\left(1+\frac{1}{K_{4}^{n_{4}}}\right)}$ (S4)

The above equation only depends on six parameters: $f_{4}, K_{4} \text{and} n_{4}$ describe GATA3 autoregulation, whereas $f_{3}, K_{3} \text{and} n_{3}$ describe GATA3 cross-regulation by ERα.. Note that we cannot solve for two unknown variables $e_{t} \text{and} g_{t}$ using a single equation. Therefore, we plug-in the ERα concentrations observed in ERα siRNA transfection experiments $(e_{t}^{obs})$ for $e_{t}$ and then use numerical optimization to find parameter sets that fit $g_{t}$ to experimentally-observed GATA3 concentrations $(g_{t}^{obs})$. In other words given $e_{t}=e_{t}^{obs}$ we minimized the following objective function for $\vec{p}=\{f_{3},K_{3},n_{3},f_{4},K_{4},n_{4}\}$:

$O_{ss}^{ER\alpha siRNA}\left(\vec{p}\right)=\sqrt{\sum_{t} \left(g_{t}-g_{t}^{obs}\right)^{2}}$ (S5)

As each of the two regulatory interactions can be positive, negative or null, we fitted the above equation to experimental data for 3^2^ or 9 different regulatory scenarios. We found that only those cases where ERα negatively regulates GATA3 (i.e. $f_{3}<1$) fit the experimental data (**Supplementary** **Figure S5, top row**). This is in agreement with the conclusions from our experimental analysis (**Figure 2**).

GATA3 siRNA

In this case we used the differential equation for ERα (normalized version of equation (1) in the main text) at quasi-steady state and followed steps analogous to those discussed above for ERα siRNA. As a result we arrived at the following equation which is analogous to equation (S4):

$\frac{1}{e_{t}}\frac{\left(1+f_{1}\frac{e_{t}^{n_{1}}}{K_{1}^{n_{1}}}\right)}{\left(1+\frac{e_{t}^{n_{1}}}{K_{1}^{n_{1}}}\right)}\frac{\left(1+f_{2}\frac{g_{t}^{n_{2}}}{K_{2}^{n_{2}}}\right)}{\left(1+\frac{g_{t}^{n_{2}}}{K_{2}^{n_{2}}}\right)}=\frac{\left(1+f_{1}\frac{1}{K_{1}^{n_{1}}}\right)}{\left(1+\frac{1}{K_{1}^{n_{1}}}\right)}\frac{\left(1+f_{2}\frac{1}{K_{2}^{n_{2}}}\right)}{\left(1+\frac{1}{K_{2}^{n_{2}}}\right)}$ (S6)

Like equation (S4) this equation also depends on six parameters: $f_{1}, K_{1} \text{and} n_{1}$ describe ERα autoregulation, whereas $f_{2}, K_{2} \text{and} n_{2}$ describe ERα cross-regulation by GATA3. Here we minimized the following objective function for $\vec{p}=\{f_{1},K_{1},n_{1},f_{2},K_{2},n_{2}\}$ given $g_{t}=g_{t}^{obs}$:

$O_{ss}^{GATA3 siRNA}\left(\vec{p}\right)=\sqrt{\sum_{t} \left(e_{t}-e_{t}^{obs}\right)^{2}}$ (S7)

Note that in this case $e_{t}^{obs}$ and $g_{t}^{obs}$ represent the ERα and GATA3 concentrations observed in GATA3 siRNA transfection experiments. Like in the previous case we fitted equation (S6) to experimental data for 9 different regulatory scenarios. We found that only those cases where GATA3 positively regulates ERα (i.e. $f_{2}>1$) fit the experimental data **(Supplementary Figure S6, bottom row**). This is also in agreement with our experimental analysis (**Figure 2**).

Taken together our model fits for ERα and GATA3 siRNA transfection experiments showed that the optimization set-up functions properly.

**Protein degradation in T47D cells**

To estimate the protein degradation rates we performed the following experiment: T47D cells were treated with 100 μg/ml cycloheximide (which blocks protein translation) and then Western blotting was used to monitor ERα and GATA3 protein levels over time (**Supplementary Figure S13A**). The data from these Western blots was first quantified using ImageJ and then fit to the equation $x=e^{-kt}$ where $x$ is the normalized protein concentration and $t$ is time, to obtain the protein degradation rates $k_{e}=0.25 h^{-1}$ and $k_{g}=0.3 h^{-1}$ (**Supplementary** **Figure S13B-C**). These degradation rates were used in all simulations.

**Table S1.** Normalized model parameters for the three network topologies in Fig. 3B-D

| **Parameter** | **Negative autoregulation** | **No autoregulation** | **Positive autoregulation** |
| --- | --- | --- | --- |
| $f_{1}$ | 0.07 | 1 | 3.78 |
| $f_{2}$ | 99.14 | 100 | 55.92 |
| $K_{1}$ | 9.81 | 0.03 | 0.33 |
| $K_{2}$ | 3.06 | 3.09 | 55.7 |
| $n_{1}$ | 3.87 | 1.56 | 2.8 |
| $n_{2}$ | 1.18 | 1.18 | 0.64 |
| $f_{3}$ | 0.1 | 0.02 | 0.12 |
| $f_{4}$ | 32.6 | 60.35 | 15.23 |
| $K_{3}$ | 1.32 | 1.32 | 1.36 |
| $K_{4}$ | 0.48 | 0.52 | 0.49 |
| $n_{3}$ | 9.7 | 10 | 8.91 |
| $n_{4}$ | 1.21 | 1.11 | 1.26 |
| $\alpha_{e}$ | 0.24 | 0.24 | 0.58 |
| $\alpha_{g}$ | 0.63 | 0.63 | 0.6 |

**Table S2.** Primer sets used for GATA3 primary transcript level measurements

| **Primer set** | **Sequences 5’ to 3’** |
| --- | --- |
| Exon1 (forward)  Intron1 (reverse) | CAGGGAGTGTGTGAACTGTGG  ATTGGAGGCTATCCTGTCAATTT |
| Exon2 (forward)  Intron2 (reverse) | CACAACCACACTCTGGAGGAG  TCCAAGCTAGGTCCTATTGCAT |

**Table S3**. Primer sets used for ChIP-qPCR

| **Position** | **Sequences 5’ to 3’** |
| --- | --- |
| ER promoter (proA) | CACATAAGGCAGCACATTAGAGAAA  GAAGACTGGGCTTAAAATAAACGC |
| ER enhancer (enh2) | GCTCTTAAGGGTTCCTGGTGG  CAGTCTTGGCCCTTTACCCA |
| GATA3 promoter | TTGGGTTGCAGTTTCCTTGT  CGACGCAACTTAAGGAGGTT |
| GATA3 enhancer | CCTCTGGGTGTTTTCAGAGC  TTACAGGGCAAAAAGGTTGG |

**
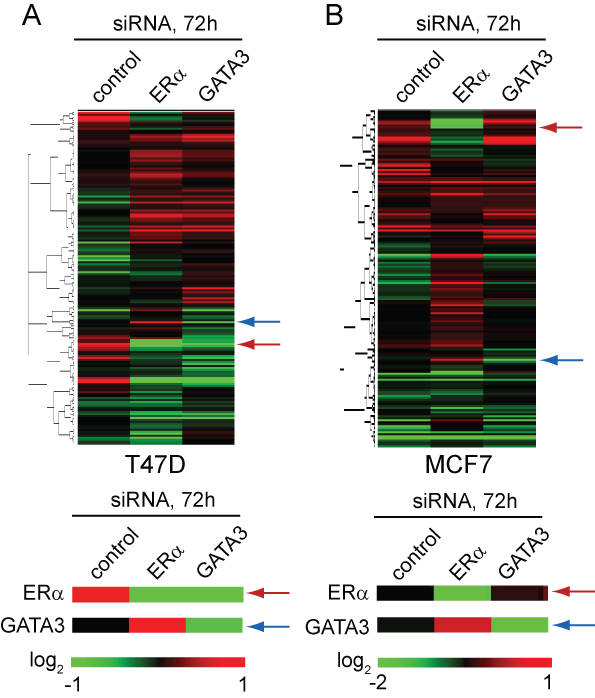
**

**Figure S1: RPPA analysis confirms asymmetric regulation between ERα and GATA3.** The fold-changes (expressed as log2 and median-centered) of ~100 cancer-related proteins, including ERα and GATA3 are shown following siRNA treatment (72h). Arrows indicate the fold-changes of ERα and GATA3 in the full RPPA dataset.

**
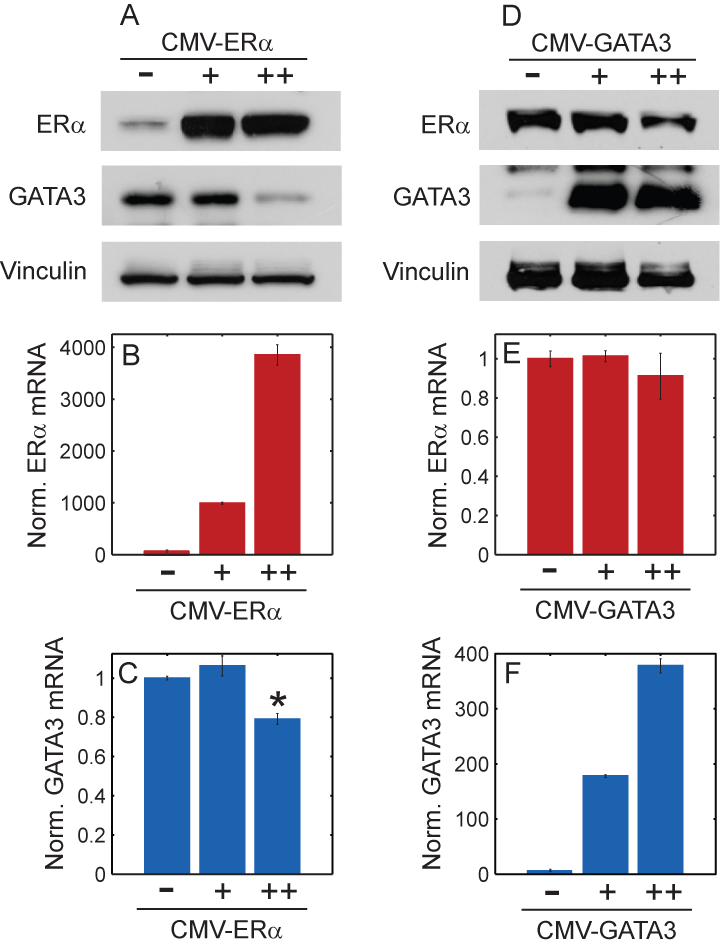
**

**Figure S2: Overexpression analysis confirms asymmetric mutual regulation between ERα and GATA3. (A)** Western blot showing protein levels in T47D cells that were transiently transfected at two different concentrations with an ERα expression vector containing the CMV-promoter. The samples were collected after 48h transfection. (**B-C)** ERα or GATA3 mRNA levels measured by qRT-PCR after ERα overexpression. Error bars represent the standard error of the mean of 3 independent experiments (Mean ± SE, n = 3). (**D)** Western blot showing protein levels in T47D cells transiently transfected at two different concentrations with a GATA3 expression vector containing the CMV-promoter. (**E-F)** ERα or GATA3 mRNA levels measured by qRT-PCR after GATA3 overexpression as in panels (**B)** and (**C)**. **p* < 0.05 by Student’s t-test.

**
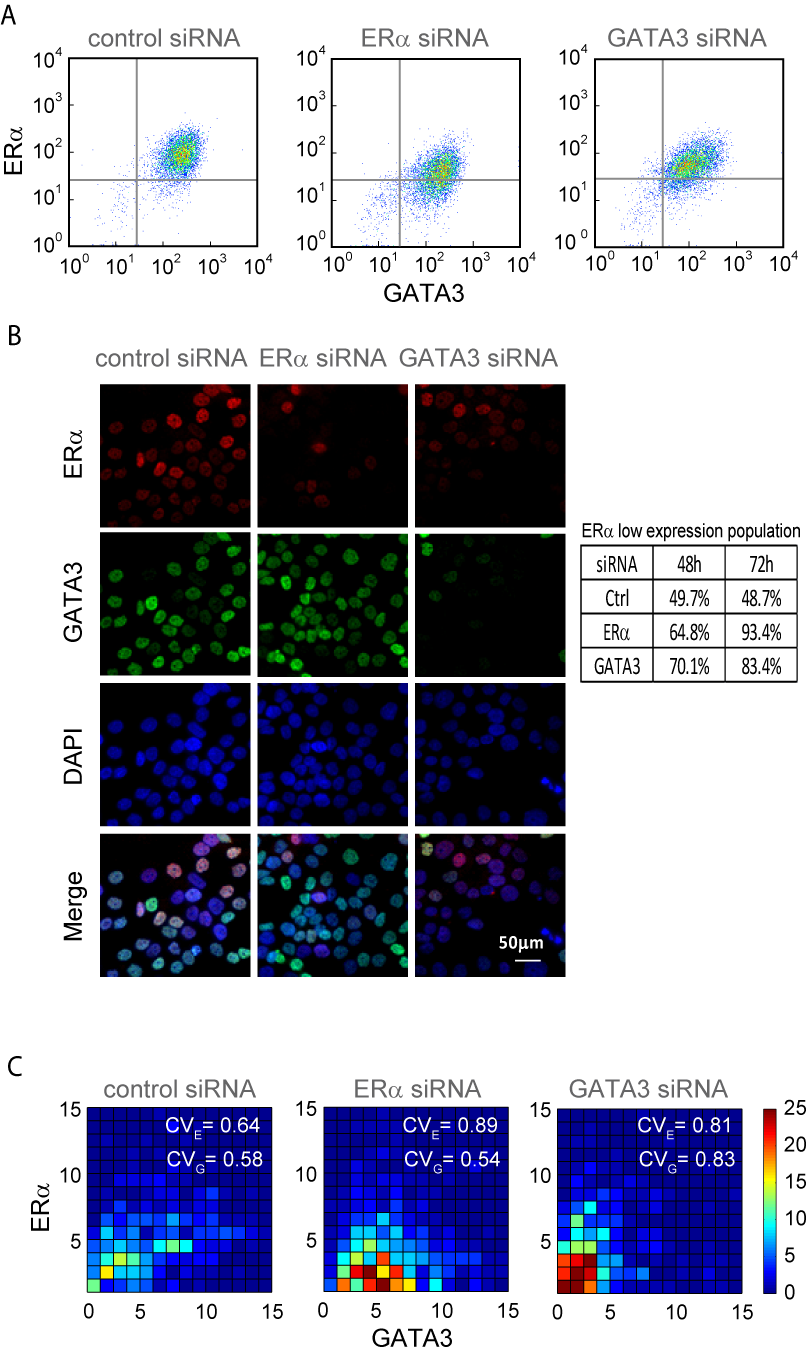
**

**Figure S3: Single cell- and single nucleus-level measurements confirm the asymmetry of regulatory connections between ERα and GATA3. (A)** Single cell level measurements of ERα and GATA3 intensities obtained by immunofluorescence labeling and flow cytometry. **(B)** Representative immunofluorescence fields of ERα and GATA3 after 48h siRNA transfection used for single nucleus-level quantification. Red is ERα, Green is GATA3, and Blue is DNA (left panel). The percentage of ERα low expression subpoplulation was indicated in the table (right panel). The median expression value was used for threshold to set the point of standard expression. **(C)** Two-dimensional probability density functions of ERα and GATA3 expression levels in single nuclei were obtained by automated image segmentation followed by averaging pixel intensities in a large number of nuclei. The color bar represents the density within the population. CV_E_ and CV_G_ represent the coefficient of variation for ERα and GATA3 expression levels respectively.


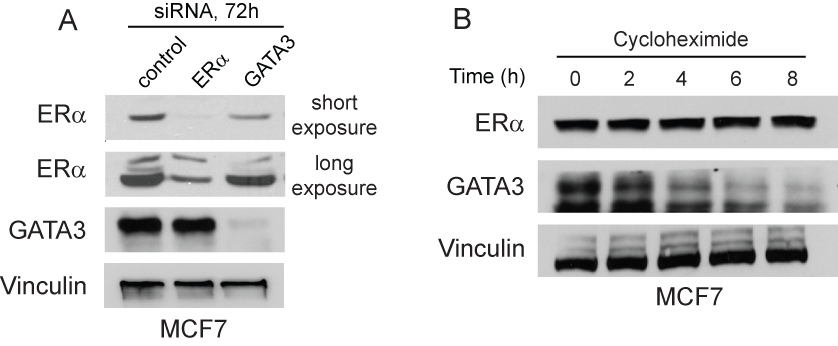


**Figure S4: ERα degradation rate is slower in MCF-7 cells compared to T47D cells.** **(A)** Western blotting of whole cell lysates from MCF-7 cells transfected with ERα or GATA3 siRNA for 72h indicates that GATA3 depletion has diminished effect on ERα levels compared to T47D cells. **(B)** Half-lives of ERα and GATA3 protein measured after CHX treatment confirm the long half-life of ERα in MCF-7 cells.


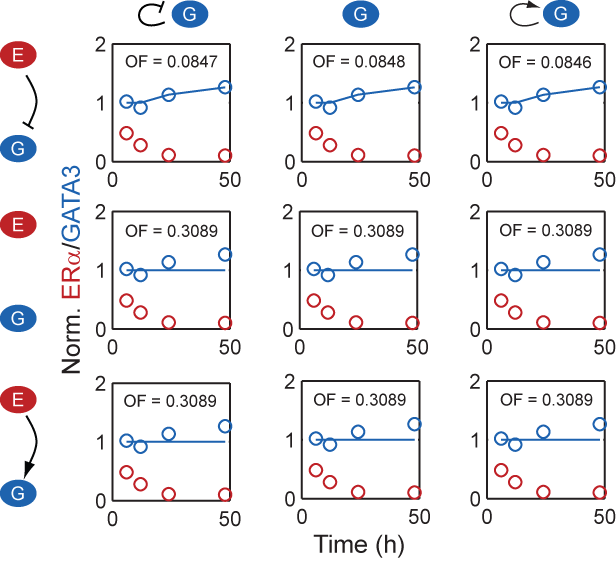


**Figure S5: Model fits for ERα  siRNA transfection experiments show that ERα negatively regulates GATA3.** Each panel shows data (ERα - red circles, GATA3 - blue circles) from the protein depletion experiment with ERα siRNA (Figure 3A) and best fit (blue line) for a unique topology. The topology for each panel is the combination of GATA3 autoregulation (noted on top of each column) and GATA3 cross-regulation by ERα (noted on left of each row). Arrowheads have the same meaning as in Figure 3.


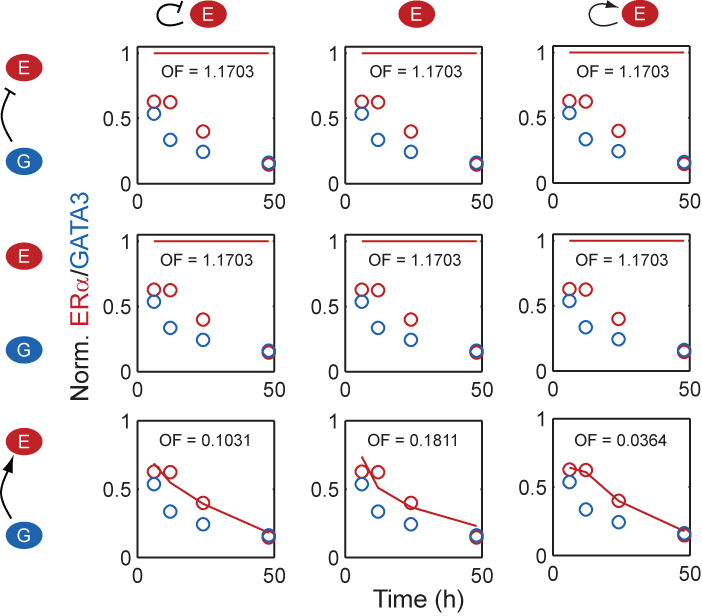


**Figure S6: Model fits for GATA3 siRNA transfection experiments show that GATA3 positively regulates ERα.** Each panel shows data (ERα - red circles, GATA3 - blue circles) from the protein depletion experiment with GATA3 siRNA (Figure 3A) and best fit (blue line) for a unique topology. The topology for each panel is the combination of ERα autoregulation (noted on top of each column) and ERα cross-regulation by GATA3 (noted on left of each row). Arrowheads have the same meaning as in Figure 3.

**
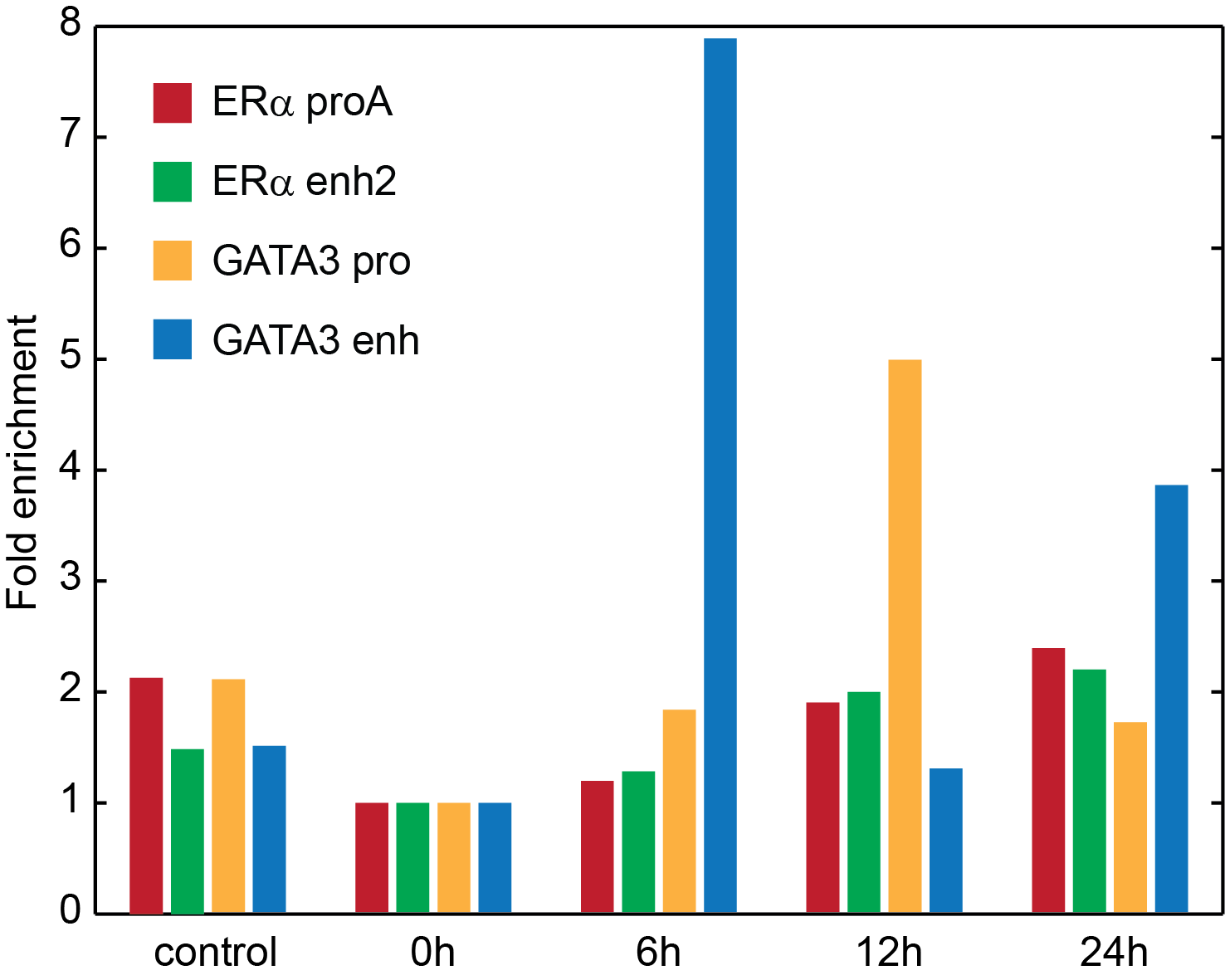
**

**Figure S7. GATA3 binds to promoter and enhancer regions of ERα and itself to regulate protein synthesis during recovery.** ChIP assay determined GATA3 recruitment to promoter and enhancer site in ERα or GATA3 gene respectively. For protein recovery, cycloheximde (CHX) was treated for 15h and then released (0h). Samples were collected at indicated time after CHX release. Control was a non-treated sample.

**
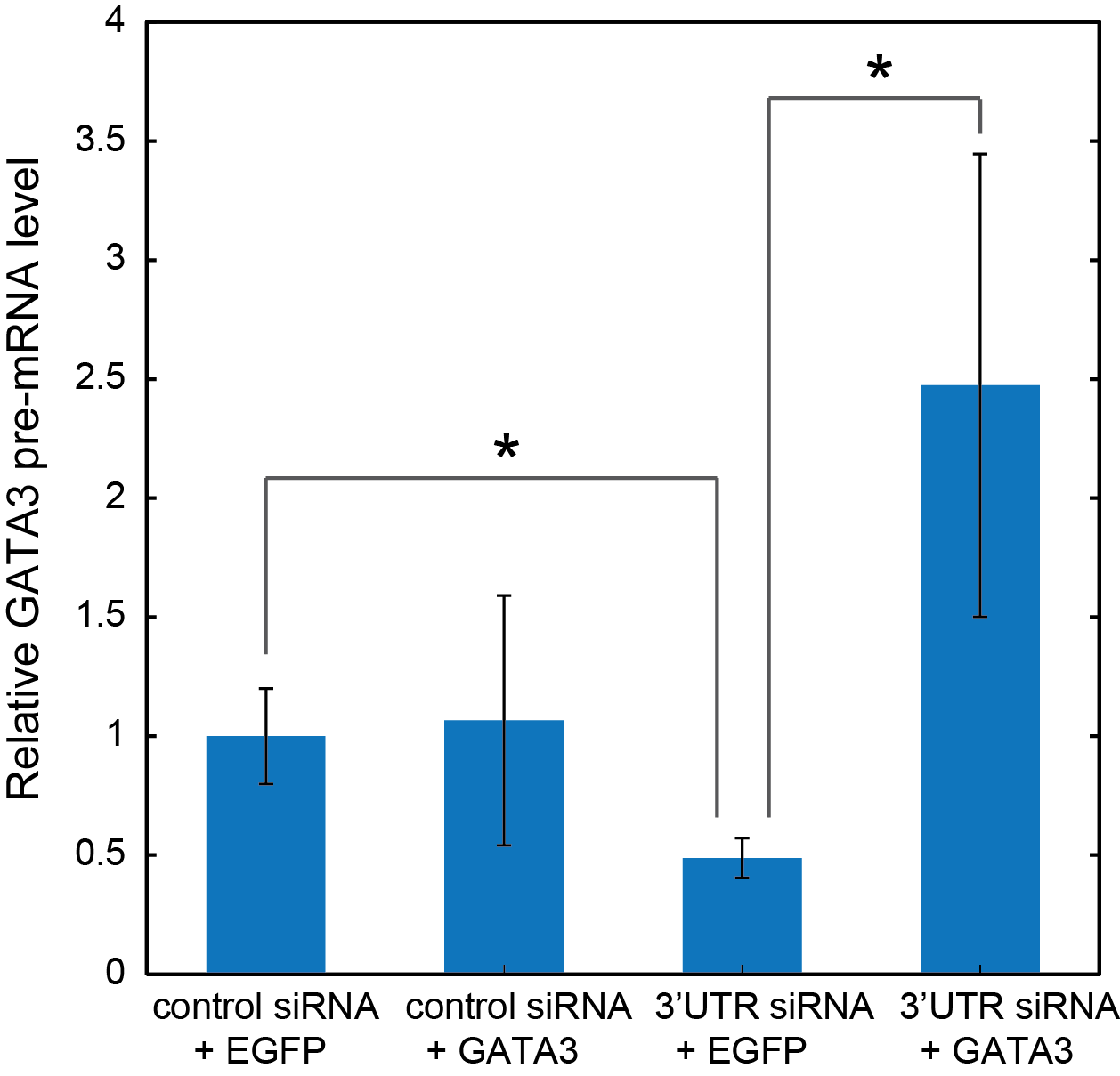
**

**Figure S8. GATA3 is under a positive autoregulation.** T47D cells were transfected with 3’UTR-targeting GATA3 siRNA or control siRNA (luciferase) for 48h, following which control (EGFP) or GATA3 cDNA containing vector was transfected. Unspliced pre-mRNA levels were measured by qRT-PCR as described in Methods (n = 4). * indicates *p* < 0.05 with Student’s *t*-test.


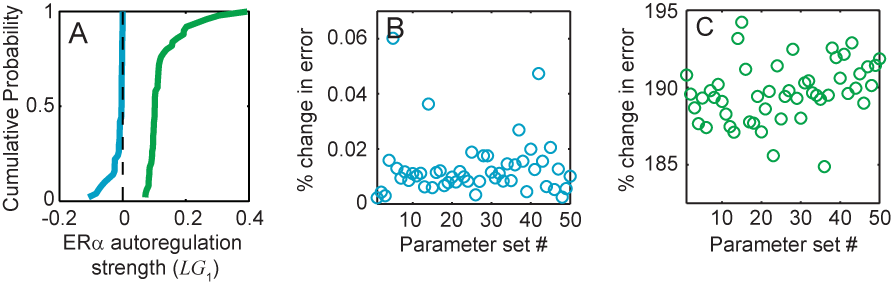


**Figure S9: Topology with ERα negative autoregulation is equivalent to no autoregulation. (A)** Cumulative probability histograms of ERα autoregulation strengths for topologies with negative (cyan) and positive (green) autoregulation. **(B-C)** Percentage change in objective function value due to removal of ERα autoregulation in the topologies with negative (cyan) and positive (green) autoregulation. For each topology the best 50 parameter sets were used for the analysis presented in panels (A-C)


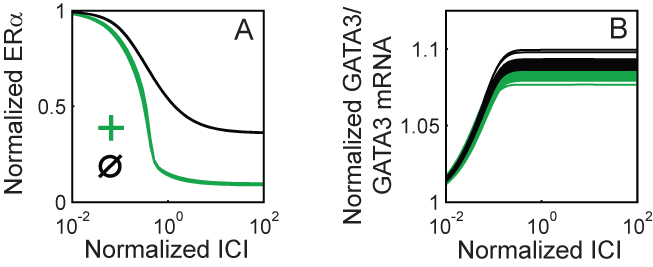


**Figure S10: Dose-response curves for protein levels are qualitatively similar for the topologies without and with positive ERα autoregulation.** Computationally generated dose-response curves depicting the change in steady-state **(A)** ERα protein **(B)** GATA3 protein and mRNA with increasing concentrations of ICI for topologies with positive (green) and without (black) autoregulation. Note that the dose-response curves for normalized GATA3 protein and mRNA are not different as the two species are proportional at steady state. For each topology the dose-response curves for best 50 parameter sets are plotted.


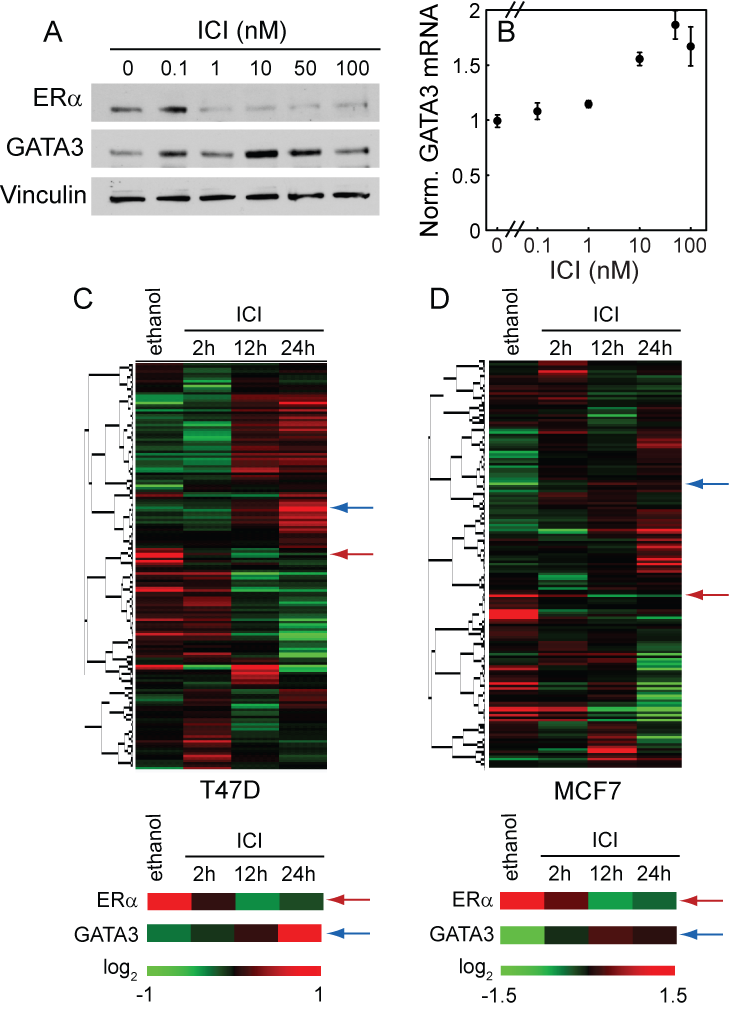


**Figure S11: ICI-mediated perturbation confirms that ERα negatively regulatesGATA3. (A)** Western blot of T47D cells cultured in serum-free medium for 48h and exposed to different concentrations of ICI for 24 hours. (**B)** GATA3 mRNA levels measured in the same conditions (ICI treatment) as in panel (a). Error bars represent the standard error of the mean of 3 independent experiments). (**C-D)** RPPA data of ~100 protein levels including those of ERα and GATA3 upon treatment with ICI. Protein samples were collected at the indicated times. Arrows indicate the expression levels of ERα and GATA3 in the RPPA clustering heat-map.

**
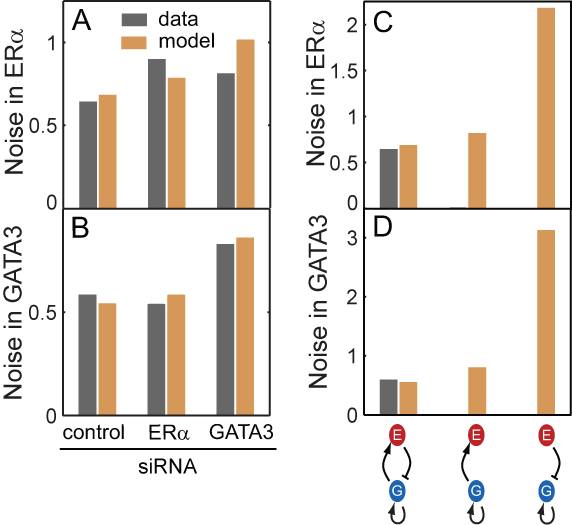
**

**Figure S12: Model with only extrinsic noise predicts that negative feedback suppresses noise in ERα and GATA3 levels. (A-B)** Experimental data (gray bars) is same as in Figure. In the model first noise was matched to the levels observed experimentally in the control siRNA case, and later the model was used to predict noise in the case of ERα or GATA3 siRNA. **(C-D)** WT bars are same as in panels A and B. Model predictions for noise in ERα and GATA3 levels in the mutants without negative feedback.


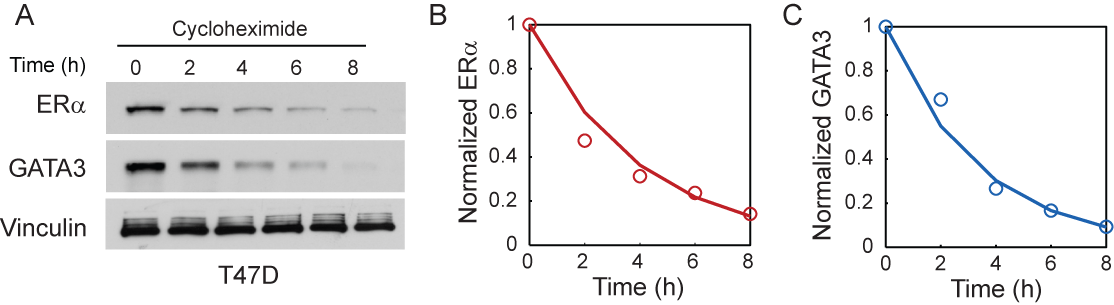


**Figure S13: Estimating ERα  and GATA3 protein degradation rates. (A)** Western blots depicting ERα and GATA3 protein levels in T47D cells that were treated with cycloheximide (representative blot, n = 3). **(B-C)** Circles represent timecourse experimental data (quantified immunoblots from panel (A)), whereas lines represent model fits that determine ERα and GATA3 protein degradation rates.

\

**References**

1. Dauvois, S., White, R. and Parker, M.G. (1993) The antiestrogen ICI 182780 disrupts estrogen receptor nucleocytoplasmic shuttling. *J Cell Sci*, **106 ( Pt 4)**, 1377-1388.

2. Reid, G., Hubner, M.R., Metivier, R., Brand, H., Denger, S., Manu, D., Beaudouin, J., Ellenberg, J. and Gannon, F. (2003) Cyclic, proteasome-mediated turnover of unliganded and liganded ERalpha on responsive promoters is an integral feature of estrogen signaling. *Mol Cell*, **11**, 695-707.

3. Elf, J. and Ehrenberg, M. (2003) Fast evaluation of fluctuations in biochemical networks with the linear noise approximation. *Genome Res*, **13**, 2475-2484.

4. Paulsson, J. (2004) Summing up the noise in gene networks. *Nature*, **427**, 415-418.

5. Ebbesen, S., Kiwitz P., Guzzella L. (2012) A generic particle swarm optimization Matlab function. *American Control Conference*, Montreal, Canada, pp. 1519-1524.
